# Mobile Phone Messaging–Based Interventions to Improve Physical Activity in Patients With Cancer: Systematic Review and Meta-Analysis

**DOI:** 10.2196/73934

**Published:** 2025-12-15

**Authors:** Xueyan Cheng, Mu-Hsing Ho, Chun Kit Chan, Denise Shuk Ting Cheung

**Affiliations:** 1 School of Nursing, Li Ka Shing Faculty of Medicine, University of Hong Kong, 3 Sassoon Road, Pok Fu Lam, Hong Kong, China, 852 39176673; 2 School of Continuing Education, Hong Kong Baptist University, Hong Kong, China

**Keywords:** mobile phone, smartphone, text messaging, telemedicine, exercise, neoplasms, systematic review

## Abstract

**Background:**

Despite the benefits of physical activity (PA) for improving cancer-related outcomes, the majority of patients with cancer fail to meet PA guidelines. Mobile phone messaging is a scalable approach for promoting PA, but its effect on improving PA among patients with cancer has not been reviewed.

**Objective:**

This review aims to systematically evaluate the effects of mobile phone messaging–based interventions in promoting PA among patients with cancer.

**Methods:**

A systematic search in 8 English and Chinese databases (PubMed, EMBASE, Web of Science, MEDLINE, the Cochrane Library, Scopus, Wanfang, and China National Knowledge Infrastructure) was performed. Randomized controlled trials that examined the effect of mobile phone messaging–based interventions on improving PA among patients with cancer were included. Potential sources of substantial heterogeneity were investigated by subgroup analysis based on participants’ characteristics, mobile phone messaging regimens, and PA estimates. Random effects models were used to estimate the overall effect size. Risk of bias was assessed by 2 independent reviewers using the revised Cochrane Collaboration’s risk of bias tool. Sensitivity analyses were performed through leave-one-out analyses, removal of outliers, and inclusion of only studies with low or some risk of bias. Potential publication bias was explored.

**Results:**

A total of 13 studies involving 777 individuals were included in this review. After intervention, mobile phone messaging–based interventions significantly improved objective PA with a small effect size (standardized mean difference [SMD]=0.37, 95% CI 0.10-0.64; *P*=.007; *I*^2^=0%), but not self-reported PA (SMD=0.20, 95% CI −0.07 to 0.47; *P*=.15; *I*^2^=56%) or step count (SMD=0.27, 95% CI −0.19 to 0.73; *P*=.25; *I*^2^=69%). Interventions that adopted more behavior change techniques and targeted patients who have completed active cancer treatment significantly improved step count. At follow-up, the effect of mobile phone messaging on self-reported PA, objective PA, and step count was found to be insignificant. Nine studies showed low or some risk of bias. Sensitivity analyses and trim-and-fill tests confirmed relatively stable effects of mobile phone messaging. No potential publication bias was identified.

**Conclusions:**

Mobile phone messaging–based interventions show promise as a scalable intervention to modestly improve objective PA in patients with cancer, though effects vary, with limited impact on self-reported PA or step count. Evidence for sustained long-term benefit remains limited, highlighting the need for rigorously designed trials with extended follow-up.

## Introduction

Abundant evidence has demonstrated that regular physical activity (PA) benefits patients with cancer by improving quality of life, enhancing aerobic fitness, supporting mental health, and reducing common treatment-related side effects [1-5]. Despite the potential benefits of PA, around 70% of patients with cancer could not achieve PA guidelines after diagnosis (ie, 150-300 min per week of moderate-intensity activity, or 75-150 min per week of vigorous-intensity activity) [6,7]. This low adherence is frequently attributed to factors inherent to the cancer experience, such as disease progression, treatment demands, and cancer-related symptoms (eg, fatigue and dyspnea) [8].

Previous interventions to promote PA among individuals with cancer have predominantly been delivered face-to-face [9]. However, this mode of delivery faces practical barriers to patient engagement, including time constraints, limited facility access, and long travel distances [10]. Over recent decades, mobile health (mHealth) technologies, such as mobile apps, wearable devices, and messaging have demonstrated potential in PA promotion among adults with cancer [11-15], providing flexibility, convenience, wide reach, and cost-effectiveness [16,17].

Mobile phone messaging facilitates communication between users via various digital platforms (eg, SMS text messaging, multimedia message service, and instant messaging), enabling the creation and real-time exchange of information. These messages can be unidirectional or interactive, standardized or tailored to individual patients, and delivered at varying frequencies [18]. Compared to other technologies, mobile phone messaging has a wider reach than web-based and app-based interventions and requires minimal digital literacy [19]. Besides, mobile phone messaging provides an effective, scalable approach for delivering behavior change techniques (BCTs), such as goal-setting, self-monitoring, and feedback, which are crucial to promote positive behavior change in patients with cancer [20].

Previous reviews have demonstrated that mobile phone messaging can improve health behaviors, including smoking cessation [21], blood pressure control [22], and weight management [23]. However, the effects of messaging on improving PA levels were inconsistent, with nonsignificant results in patients with type 2 diabetes [24] and significant results in general adult populations [25]. While several reviews have explored the effect of broader eHealth or mHealth modalities on PA in populations with cancer [11-15], none have specifically evaluated the effect of mobile phone messaging–based interventions. Therefore, this study aims to synthesize existing evidence and estimate the overall effect of mobile phone messaging–based interventions for promoting PA in patients with cancer.

## Methods

### Search Strategy

A thorough review of the literature was performed using PubMed, EMBASE, Web of Science, MEDLINE, the Cochrane Library, Scopus, and key Chinese databases, including Wanfang and China National Knowledge Infrastructure, from the inception of databases to February 2025.

Search terms included:

(“cancer” OR “cancer survivors” OR “neoplasms”) AND (“lifestyle intervention” OR “physical activity” OR “exercise” OR “behavior change”) AND (“message” OR “messaging” OR “text message” OR “text messaging” OR “mobile message” OR “mobile phone messaging” OR “short message service” OR “SMS” OR “instant message”) AND (“randomized controlled trial” OR “RCT” OR “clinical trial” OR “placebo” OR “randomized” OR “randomly” OR “trial”).

In addition, the reference lists in published reviews and meta-analyses were examined to identify papers that the electronic search missed. We also performed a search in gray literature, including the preprint studies in the 8 databases, and in OpenGrey, ProQuest Dissertations & Theses, and Electronic Theses and Dissertations (EBSCO Open Dissertations). The research strategy is shown in Multimedia Appendix 1.

This review follows the PRISMA (Preferred Reporting Items for Systematic Reviews and Meta-Analyses) guidelines (Checklist 1) [26] to ensure a methodical approach to data collection and analysis. The review has been registered at PROSPERO (CRD42024557519), and there were no deviations from the registered protocol.

### Eligibility Criteria

The inclusion criteria were as follows: (1) the study design should be randomized controlled trials (RCTs), including both full and pilot trials; (2) participants should be adults (≥18 y) with cancer; (3) the intervention must primarily use mobile phone messaging as the main or sole delivery channel of delivering the intervention’s content, instructions, or engagement with participants. In the case of broader interventions, mobile phone messaging must be a central and indispensable element, contributing significantly to the intervention’s intended effect (eg, accounting for ≥50% of communication touch points or engagement time, as reported in the study; (4) outcomes should include PA estimates, such as moderate-to-vigorous PA (MVPA), total PA, and step count; (5) the control or comparison condition should not involve the delivery of messaging; and (6) only studies published in English and Chinese were included. There were no restrictions on the type of cancer, treatment status, or the form of PA. Interventions where messaging is a minor or supplementary component (eg, used only for reminders or scheduling rather than delivering core content) were excluded to maintain focus on messaging-driven interventions.

### Study Selection

All records retrieved from the databases were imported into EndNote (Clarivate), and the software’s built-in duplicate identification function was used to remove duplicates. The titles and abstracts of the retrieved papers were independently screened and cross-checked by 2 reviewers (XC and CKC). The full-text studies were obtained and assessed for eligibility against the inclusion criteria by the 2 reviewers independently. Discrepancies were resolved through discussion or, if necessary, adjudication by a third investigator (DSTC). When multiple studies describing the same RCT were identified, the study presenting the primary and most comprehensive results was selected for review.

### Data Extraction and Quality Assessment

Relevant data were extracted independently by XC and CKC. Information extracted included participant characteristics (eg, country, cancer types, mean age, gender, and cancer treatment status) and study characteristics (eg, intervention components, control conditions, intervention duration and frequency, PA estimates, theories, number of BCTs [27], and adverse events [AEs]). Regarding BCTs extraction, the standardized BCT Taxonomy v1 [27], a widely accepted framework for identifying and coding intervention components, was used for reliable identification. Data on baseline, after intervention, and follow-up PA estimates (mean and SD or SE or 95% CI), or change in PA estimates from baseline (mean change and SD) were extracted. For trials with more than 2 arms, only data from the relevant intervention group and the control group were extracted.

Risk of bias was assessed by 2 independent reviewers (XC and CKC) using the revised Cochrane Collaboration’s risk of bias tool (Risk of Bias 2) with a Microsoft Excel template [28] on the domains of (1) bias arising from the randomization process, (2) bias due to deviations from intended interventions, (3) bias due to missing outcome data, (4) bias in measurement of the outcome, and (5) bias in selection of the reported result. Assessments were cross-checked, and any discrepancies were resolved through discussion or, if needed, adjudication by a third reviewer (DSTC).

A grade of recommendation, assessment, development, and evaluation (GRADE) approach was adopted using an online GRADEpro tool to assess the confidence of intervention effects [29]. The assessed domains included risk of bias, inconsistency, indirectness, imprecision, and publication bias.

### Data Synthesis and Statistical Analyses

All analyses of pooled effects were performed using the *meta* package in R (version 4.3.0; R Foundation for Statistical Computing). Forest plots were used to display the results of individual studies and syntheses. Overall effect sizes were calculated to pool the study results on the standardized mean difference (SMD) of the change in self-reported PA level, objective PA level, and step count between the intervention and control group at postintervention and follow-up. An SMD of 0.2, 0.5, and 0.8 corresponds to small, medium, and large effect sizes, respectively. Primarily, SMD was derived from the mean difference and SD of change. For studies that did not report mean change and SD, SMD was estimated from baseline and postintervention or follow-up values of mean and SD, whereas the SD of change was imputed based on a correlation coefficient (*r*) derived from the only included study that presented baseline and post-intervention means, SDs, and change (ie, *r*=0.68) [30]. These methods of calculation were in line with the Cochrane handbook for imputing data for SMD in systematic reviews [31]. For studies that only provided median and IQR for PA levels, mean and SD were calculated based on Luo et al [32] and Shi et al [33]. A positive SMD within the meta-analysis indicated an increased level of PA for intervention groups compared with control groups. A random effects model was used for all outcomes.

Heterogeneity was investigated in each analysis using *I*^2^ values that range from 0% to 100%, with higher values indicating greater heterogeneity. Heterogeneity greater than 50% was considered substantial [34]. Potential sources of substantial heterogeneity were investigated by subgroup analysis of treatment status (posttreatment vs other status); cancer types (mixed vs single cancer); intervention period (≤3 mo vs >3 mo); message frequency (daily vs less than daily); interactive message (yes vs no); tailored messages (yes vs no), including a wearable device in the intervention (yes vs no); theory basis (yes vs no); and adoption of less or more than the median number of BCTs of studies in this review. Sensitivity analyses were performed through leave-one-out analyses, removal of outliers, and inclusion of only studies with low or some risk of bias [1]. We also assessed publication bias using funnel plots and the Egger linear regression method, with *P*<.05 taken as an indication of publication bias [35].

## Results

### Systematic Review: Selection Results

**Figure 1.**
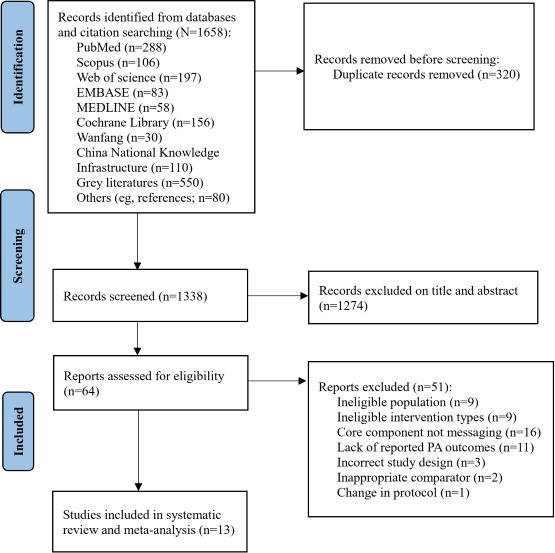
PRISMA (Preferred Reporting Items for Systematic Reviews and Meta-Analyses) flow chart. PA: physical activity (adapted from Page MJ, et al [26] and published under Creative Commons Attribution 4.0 International License [36])

Figure 1 shows the study selection process. The search of all databases and identification through other sources resulted in a total of 1658 records. Following the removal of duplicates, the total was 1338 records. We excluded 1274 records based on titles and abstracts. Therefore, a total of 64 studies were assessed for eligibility. Fifty-one studies were excluded after applying the inclusion criteria. The primary reasons for exclusion were the absence of mobile phone messaging as a core component (n=16), a lack of reported PA outcomes (n=11), and an ineligible population (n=9). Other reasons included an ineligible intervention type (n=9), an incorrect study design (n=3), an inappropriate comparator (n=2), and a change in the study protocol (n=1). Subsequently, 13 studies met the criteria for the systematic review and meta-analysis. During the selection process, one discrepancy was resolved by a third adjudicator (DSTC), resulting in the exclusion of a study because mobile phone messaging was not a core intervention component. No eligible study was identified from the Chinese databases. All studies were published between 2018 and 2023, and they were based in 4 countries: the United States (n=9, 69%), Australia (n=2, 15%), France (n=1, 8%), and Ireland (n=1, 8%*).*

Table 1 summarizes participant characteristics of the included studies. A total of 777 participants from the 13 included studies were included for this review. These studies encompassed multiple cancer types, with breast and prostate cancer being the most prevalent. Specifically, 5 (38%) studies focused on mixed cancer types [30,37-40], 3 (23%) focused on breast cancer [41-43], 1 (8%) investigated endometrial cancer [44], 1 (8%) examined lung cancer [45], 2 (15%) studied prostate cancer [46,47], and 1 (8%) focused on colon or rectal cancer [48]. Four (31%) studies involved solely female patients [41-44], and 2 (15%) only involved male patients [46,47]. The mean ages of the participants ranged from 49.7 (SD 13.7) to 69.8 (SD 8.7) years. Regarding the status of cancer treatment, 69% (9/13) studies involved participants who had completed active cancer treatment, 2 (15%) recruited patients receiving concurrent cancer treatment [39,45], 1 (8%) recruited patients scheduled for treatment [42], and 1 (8%) did not specify or restrict treatment status [47]. Cancer stage was only reported in 8 (62%) studies: 3 (23%) limited to patients with stage I to III cancer [30,42,46], 2 (15%) included patients with stage 0 to III cancer [40,43], 1 (8%) aimed at patients with stage III or IV cancer only [45], 1 (8%) focused on stage II-III cancer [48], and 1 (%) recruited patients at stage I to IV [47]. Five (38%) studies did not report the cancer stages of the participants.

**Table 1.** Participant characteristics of the included studies.

Author, year	Country	Sample size	Age (y), mean (SD)	Female proportion (%)	Cancer type (treatment status)
Allicock et al, 2021 [41]	The United States	n=22 IG^a^:CG^b^=13:9	52.2 (9.2)	100	Breast cancer (≥6 mo since completion of treatment)
Bade et al, 2021 [45]	The United States	n=40 IG:CG=20:20	64.9 (8.7)	75	Stage III/IV nonsmall cell lung cancer (at any stage of treatment)
Gell et al, 2020 [30]	The United States	n=66 IG:CG=34:32	61.4 (9.0)	83	Stage I-III mixed cancer (completion of oncology rehabilitation)
Gomersall et al, 2019 [38]	Australia	n=36 IG:CG=18:18	64.8 (9.6)	36	Mixed cancer (at least 1 mo postsurgery)
Haggerty et al, 2017 [44]	The United States	n=21 IG:CG=11:10	62.2 (8.7)	100	Endometrial cancer (no current and planned treatment)
Kenfield et al, 2019 [46]	The United States	n=60 IG:CG=30:30	64.8 (6.2)	0	Stage T1-T3a prostate cancer (completion of treatment ≥3 mo)
Singleton et al, 2023 [43]	Australia	n=156 IG:CG=78:78	54.8 (10.9)	100	Stage 0-III breast cancer (within 18 mo of finishing active treatment)
Van Blarigan et al, 2019 [48]	The United States	n=39 IG:CG=20:19	54.0 (11.0)	59	Stage II-III colon or rectal cancer (completion of treatment ≥3 mo and <2 y)
Villaron et al, 2018 [39]	France	n=43 IG:CG=21:22	49.7 (13.7)	72	Mixed cancer (currently undergoing treatment)
Walsh et al 2021 [37]	Ireland	n=123 IG:CG=62:61	57.4 (8.0)	74	Mixed cancer (active treatment completed)
SenthilKumar et al, 2024 [42]	The United States	n*=*44 IG:CG=22:22	57.0 (9.5)	100	Stage I-III breast cancer (scheduled treatment)
Chan et al, 2020 [47]	The United States	n*=*99 IG:CG=50:49	69.8 (8.7)	0	Stage I-IV prostate cancer (no restriction on treatment status)
Hassoon et al, 2021 [40]	The United States	n*=*28 IG:CG=14:14	62.1 (9.8)	90	Stage 0-III mixed cancer (completion of treatment for at least 3 mo)

a IG: intervention group.

b CG: control group.

### Study Characteristics

#### Treatment Conditions

Table 2 summarizes the study characteristics. Regarding message content, 7 (54%) studies only included PA in an intervention content [30,37-40,45,48], while 6 (46%) consisted of PA plus other components, including 5 (38%) with diet [41-44,47], and 1 (8%) with both nutrition and smoking cessation information [46]. The lengths of the interventions ranged from 1 to 6 months. The majority of studies adopted wearable devices (10/13, 77%). Some studies included in-person interaction (coaching about PA and supervised exercise sessions; 6/13, 46%), educational materials (3/13, 23%), or ecological momentary assessment about daily PA (1/13, 8%). Regarding the frequency of message sending, 5 (38%) studies reported daily messaging, and 8 (62%) studies reported weekly or less frequent messaging. Four (31%) studies used SMS text messaging to deliver messages [30,37,39,43], 2 (15%) used a multimedia platform Propelo [38] and Sense Health [44], 2 (15%) used an app (Health Information Portability and Accountability Act [HIPAA] Compliant Texting, and myTapp) [42,45], 1 (8%) used artificial intelligence agent [41], and the other 4 (31%) studies did not report the platform. Three (23%) studies used interactive messages [38,44,46], and the other 10 (77%) used nonresponse messages. Six (46%) studies used tailored messages [30,37,38,40,41,45], and the other 7 (54%) used nontailored messages. Control group design for all studies used positive or passive groups. Positive control included health education, accelerometers, and standard clinical exercise rehabilitation programs [30,37-42,47,48], while passive studies included usual care [43-46]. The median number of BCTs used was 10 (IQR 8.5-13.5; Multimedia Appendix 2), including 7 (54%) studies adopting ≥10 BCTs [30,37,38,40,42,46,47] and 6 (46%) studies <10 BCTs [39,41,43-45,48]. Besides, 7 out of 13 (54%) interventions were developed based on theories, including social cognitive theory [28,40,41,46], theory of planned behavior [46,48], and health belief theory [40] (Multimedia Appendix 3).

**Table 2.** Study characteristics of the included studies.

Author, year	Intervention group	Message content	Control group	Intervention length, frequency of messages	Data collection points	PA^a^ tracker	PA goals	PA measurement	Theory basis	Adverse events
Allicock et al, 2021 [41]	Tailored, noninteractive text messages +EMA^b^ with mobile app	PA and diet (feedback on daily diet and exercise)	Positive control: EMA with mobile app	1 month, daily	Baseline, 4 weeks, and 8 weeks	No	N/A^k^	Self-reported PA: MVPA^d^ (The Behavioral Risk Factor Surveillance System PA questionnaire) Objective PA: Total PA (ActiGraph)	Social cognitive theory and control theory	Not reported
Bade et al, 2021 [45]	Nontailored, noninteractive text messages via an app	PA (step count goal+individual step count)	Passive control: usual care	3 months, twice daily	Baseline, 12 weeks	Yes (Fitbit)	Individualized goals based on patient’s average daily step count during Week 1 (adding 400 steps per day to the average daily step count)	Self-reported PA: MVPA (The Modifiable Activity Questionnaire)	N/A	4 serious (ie, hospitalization, fall) and 2 minor adverse events (ie, ankle pain and bronchitis) unrelated to the study
Gell et al, 2020 [30]	Tailored, noninteractive text messages via SMS	PA (PA intentions, barriers, short-term goals, and measured PA levels)	Positive control: Fitbit only	2 months, 25 text messages in total	Baseline, 8 weeks	Yes (Fitbit)	Self-directed goal setting (informed by current level of PA and recommended guidelines)	Objective PA: MVPA (Fitbit)	Social cognitive theory	Not reported
Gomersall et al, 2019 [38]	Tailored, interactive text messages via a multimedia platform+4-week standard clinical exercise rehabilitation program same as control	PA (included a minimum of 2 educational tips, 3 real-time prompts, and 1 goal check text per fortnight)	Positive control: 4-week standard clinical exercise rehabilitation program	3 months, ≥6 text messages per fortnight	Baseline, 4 weeks, and 12 weeks	No	4×40 mins each week	Self-reported PA: MVPA (adult version of the Multimedia Activity Recall for Children and Adults) Objective PA: MVPA (activPAL)	N/A	One overbalanced and fall during lung exercise
Haggerty et al, 2017**^e^** [44]	Tailored, interactive text messages via a multimedia platform	PA and dietary (feedback, support, prompting, quiz items, and strategies to adhere to PA and diet behaviors)	Passive control: usual care	6 months, daily	Baseline and 6 months	No	Moderate PA, starting from 50 minutes per week, increases to 175 minutes per week	Self-reported PA: total PA (International Physical Activity Questionnaire Short Form)	N/A	No adverse events
Kenfield et al, 2019 [46]	Nontailored, interactive text messages	PA, diet, and smoking cessation recommendations	Passive control: usual care	3 months, 4‐5 text messages per week	Baseline and 12 weeks	Yes (Fitbit)	Personalized recommendations	Self-reported PA: MVPA (self-designed PA questionnaire) Objective PA: MVPA Step count (Actigraph)	Theory of planned behavior	25 in the intervention group and 18 in the control group reported 89 nonserious adverse events related to PA (ie, low back pain, knee pain, and arthritis)
Singleton et al, 2023 [43]	Nontailored, noninteractive text messages via SMS	PA and healthy diet (PA and healthy diet, social and emotional well-being, and general breast cancer info)	Passive control: usual care	6 months, 4 text messages per week	Baseline and 6 months	No	N/A	Self-reported PA: total PA (Global Physical Activity Questionnaire)	N/A	Not reported
van Blarigan et al, 2019 [48]	Nontailored, noninteractive text messages via SMS+print material	PA (benefits of PA; prompts for goal setting or planning, advice and tips for incorporating activity into daily life, and challenges and quizzes to increase engagement)	Positive control: print material	3 months, daily	Baseline and 12 weeks	Yes (Fitbit)	To meet WHO^f^ recommendations (150 moderate PA or 75 Vigorous PA+twice to three times RT^g^ weekly)	Objective PA: MVPA (Fitbit) Step count (Fitbit)	Theory of planned behavior	20 in the intervention group, 21 in the control group reported 75 nonserious adverse events (ie, low back pain, and knee pain) relating to PA
Villaron et al, 2018 [39]	Nontailored, noninteractive text messages via SMS + pedometer	PA (recommendations to increase PA)	Positive control: pedometer only	2 months, weekly	Weeks 1 to 8 (Weekly)	Yes (pedometer)	N/A	Step count (pedometer)	N/A	Not reported
Walsh et al, 2021 [37]	Tailored, noninteractive text messaging via SMS	PA (feedback on average daily step count and a goal of increasing step count)	Positive control: standard care + Fitbit	3 months, weekly	Weeks 1 to 24 (weekly)	Yes (Fitbit)	Personalized goal	Self-reported PA: total PA (Goldin Leisure-Time Exercise Questionnaire) Step count (Fitbit)	N/A	Not reported
SenthilKumar et al, 2024 [42]	Nontailored, noninteractive text messaging via an app	PA and diet (social support and reinforce adherence to exercise and diet)	Positive control: diet or exercise information binder + Fitbit	12 weeks, 3 times per week	Baseline, week 12, and week 24	Yes (Fitbit)	To engage in 150 minutes of moderate PA and 2 sessions of resistance exercise weekly	Self-reported PA: total PA (Godin Leisure Physical Activity survey)	Social cognitive theory	No adverse events
Chan et al, 2020^h^ [47]	Nontailored, noninteractive text messaging	PA and diet (support to reinforce exercise and diet)	Positive control: website education	12 weeks, 4 texts per week	Baseline, week 12, and week 24	Yes (Fitbit)	Personalized goal	Self-reported PA: MVPA (The Community Health Activities Model Program for Seniors Survey)	Social cognitive theory	15 in the intervention group and 8 in the control group reported nonserious adverse events (ie, joint pain, bone pain, and muscle pain)
Hassoon et al,^j^ 2021 [40]	Tailored, noninteractive text messaging via AI-agent^i^+Fitbit	PA (AI-based contents to increase PA)	Positive control: Printed written information	4 weeks, three messages per day	Baseline, week 4	Yes (Fitbit)	10,000 steps per day	Step count (Fitbit)	Health belief theory	No adverse events

a PA: physical activity.

b EMA: ecological momentary assessment.

c N/A: not applicable.

d MVPA: moderate-to-vigorous physical activity.

e An arm on 16-week phone counseling sessions was excluded due to lack of messaging component.

f WHO: World Health Organization.

g RT: Resistence Training

h An arm on combination of website and personalized diet and exercise prescription was excluded due to lack of messaging component, and an arm on combination of website education, personalized diet and exercise prescription, Fitbit, text messages, and two 30-min phone calls was excluded because messaging is not the core component.

i AI: artificial intelligence.

j An arm on AI-based voice intervention was excluded due to lack of messaging component.

#### Adverse Events

Table 2 summarizes the reporting of AEs. Three (23%) studies reported no AEs [40,42,44]. Three (23%) studies reported nonserious AEs related to PA in both intervention and control groups, such as low back pain, knee pain, inflammation of the joints, arthritis, and joint pain [46-48]. One (8%) study reported an adverse event of falling during exercise [38]. One (8%) study reported a single AE of falling during exercise [38]. Another study reported 4 serious AEs (chronic obstructive pulmonary disease exacerbation, pneumonia, and hyperthyroidism) and 2 minor AEs (ankle pain and bronchitis), all unrelated to the intervention [45]. Six (46%) studies did not mention the presence or absence of AEs.

#### Outcomes and Measurement

The outcome measures used by the studies are listed in Multimedia Appendix 4. Five (38%) studies reported objective PA outcomes, with 4 (31%) measuring MVPA and 1 (8%) assessing total PA. All studies measured objective PA using accelerometers (ie, FitBit and activPAL) [30,38,41,46,48]. Self-reported PA was reported by 9 (69%) studies, with 5 (38%) in terms of MVPA and 4 (31%) in terms of total PA. Self-reported questionnaires included the Behavioral Risk of Factor Surveillance System Physical Activity Questionnaire [41], Modified Activity Questionnaire [45], Adult version of Multimedia Activity Recall for Children and Adult [38], International Physical Activity Questionnaire Short Form [44], Global Physical Activity Questionnaire [43], investigator-designed PA questionnaire [46], Goldin Leisure-Time Exercise Questionnaire [37,42], and the Community Health Activities Model Program for Seniors Survey [47]. Step count was reported by 5 (38%) studies [37,39,40,46,48] and measured using accelerometers (ie, activPAL and Actigraph) except 1 (8%) study which used pedometers [39]. Five (38%) studies reported the use of more than 1 PA measurement [37,38,41,46,48]. Four (31%) studies included a follow-up time point in addition to postintervention, including 4 weeks and 12 weeks after the end of intervention [37,41,42,47].

#### Risk of Bias and Certainty of Intervention Effects

**Figure 2.**
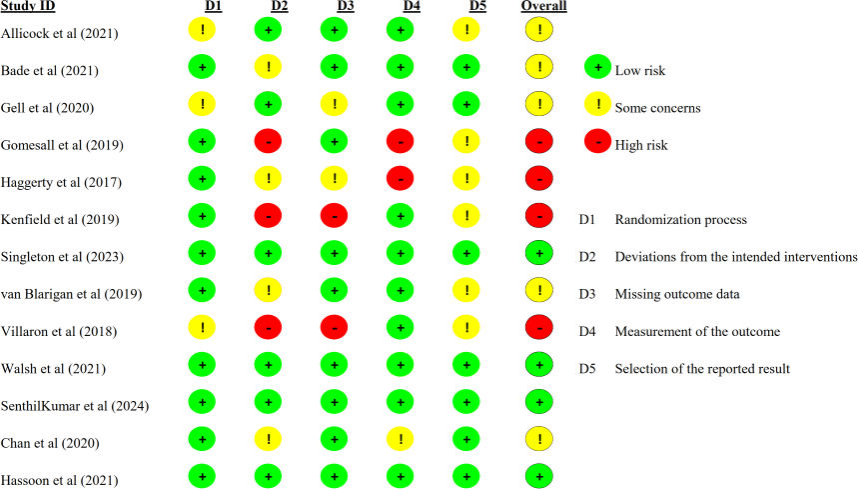
Risk of bias of included studies [30,37-48].

The assessed quality of the included studies is shown in Figure 2. In general, 4 (31%) RCTs showed low risk of bias, 5 (38%) studies were at some risk of bias, while 4 (31%) RCTs showed a high risk of bias. All studies reported the randomization process with low or some risk of bias, including 10 (77%) with low risk of bias and 3 (23%) with some concerns for a lack of reporting on allocation concealment or with baseline difference. Three (23%) RCTs reported a high risk of bias in deviations from the intended interventions. This was primarily due to a lack of blinding of participants and personnel, combined with the reporting of outcome analyses that were not based on the “intention-to-treat” principle, potentially introducing performance and analytic bias. Two (15%) reported a high risk of bias due to substantial dropout, and without any evidence that the result was not biased by missing outcome data. Two (15%) had a high risk of bias in the measurement of the outcome, because the outcome assessors were not blinded to the intervention. Seven (54%) studies reported a low risk of bias in the selection of the reported results, while 6 (46%) had some concerns due to the lack of a prespecified analysis plan.

The GRADE assessment indicated that the certainty of evidence for the effect of text messaging on PA was moderate for objective PA postintervention, step count postintervention, very low for self-reported PA at postintervention, and moderate for self-reported PA at follow-up (Multimedia Appendix 5).

### Meta-Analysis

#### Meta-Analysis at Postintervention for Objective PA Levels

**Figure 3.**
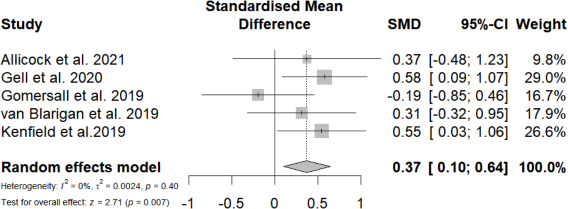
Overall standardized mean difference of mobile phone messaging–based interventions on objective physical activity levels at postintervention [30,38,41,46,48].

A total of 5 (38%) studies reported the effects of mobile phone messaging–based interventions on objective PA levels at postintervention. Mobile phone messaging–based interventions had a statistically significant yet small effect in improving objective PA levels (SMD=0.37, 95% CI 0.10-0.64; *P*=.007; *I²*=0%; Figure 3).

#### Meta-Analysis at Postintervention for Self-Reported PA Levels

**Figure 4.**
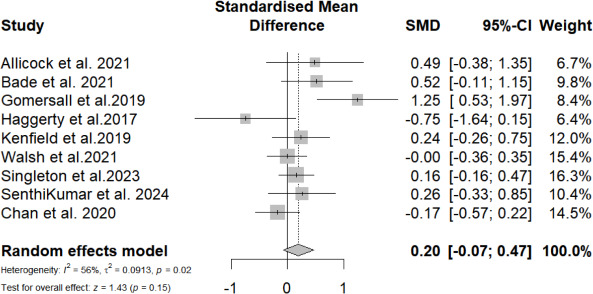
Overall standardized mean difference of mobile phone messaging–based interventions on self-reported physical activity levels at postintervention [37,38,41-47].

A total of 9 (69%) studies reported the effects of mobile phone messaging–based interventions on self-reported PA levels at postintervention. The overall SMD of mobile phone messaging–based interventions in improving self-reported PA level was not statistically significant (SMD=0.20, 95% CI −0.07 to 0.47; *P=*.15) with relatively high heterogeneity (*I*^2^=56%; *P*=.02; Figure 4). All participant and intervention characteristics showed no statistically significant difference between groups.

#### Meta-Analysis at Postintervention for Step Count

**Figure 5.**
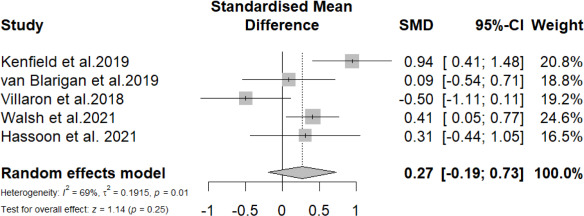
Overall standardized mean difference of mobile phone messaging–based intervention on step count at postintervention [37,39,40,46,48].

A total of 5 (38%) studies reported the effects of mobile phone messaging–based interventions on step count among patients with cancer at postintervention. The overall SMD of mobile phone messaging–based interventions in improving step count was not statistically significant (SMD=0.27, 95% CI −0.19 to 0.73; *P=*.25) with relatively high heterogeneity (*I*^2^=69%; *P=*.011; Figure 5). Subgroup analyses showed no statistical effects on heterogeneity for all variables except treatment status and number of BCTs (test for subgroup difference *P*=.007 and *P*=.03, respectively). Specifically, studies targeting posttreatment patients showed a statistically significant effect in improving PA levels (4/5, 80%; SMD=0.46, 95% CI 0.12-0.80; *P=*.007; *I*^2^=37%) and nonsignificant effect for patients under treatment (1/5, 20%; SMD=−0.50, 95% CI −1.11 to 0.11). Interventions that adopted ≥10 BCTs showed statistically significant effect (3/5, 60%; SMD=0.56, 95% CI 0.19-0.93; *P=*.003; *I*^2^=35%), while those adopting less than 10 BCTs reported nonsignificant effect (2/5, 40%; SMD=−0.21, 95% CI −0.79 to 0.36; *P=*.47; *I*^2^=42%; Multimedia Appendix 6).

#### Meta-Analysis at Follow-Up for Self-Reported, Objective PA Levels, and Step Count

**Figure 6.**
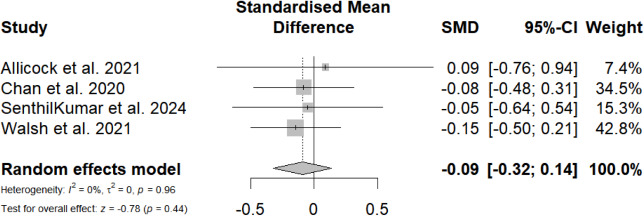
Overall standardized mean difference of mobile phone messaging–based interventions on self-reported physical activity levels at follow-up [37,41,42,47].

Four (31%) studies reported the effects of mobile phone messaging–based interventions on self-reported PA levels at follow-up. The overall SMD was not statistically significant (SMD=−0.09, 95% CI −0.32 to 0.14; *P=*.44), with zero heterogeneity (Figure 6). Only 1 out of 4 (25%) studies reported the effect of mobile phone messaging–based interventions on objective PA levels and step count, respectively, both of which showed nonsignificant effects (SMD=0.58, 95% CI −0.29 to 1.45; SMD=0.23, 95% CI −0.12 to 0.59).

#### Sensitivity Analyses

Sensitivity analyses results are presented in MultimediaAppendices 7^10^. In leave-one-out analyses at postintervention, mobile phone messaging interventions’ effects on self-reported PA remained nonsignificant (Multimedia Appendix 7). Step count effects became significant after omitting the Villaron study (SMD=0.46, 95% CI 0.12-0.80; *P*=.007; *I*²=37%; Multimedia Appendix 8). Objective PA effects were robust when excluding the studies by Allicock et al [41], Gomersall et al [38], or van Blarigan et al [48], but became nonsignificant after removing studies by Gell et al [30] or Kenfield et al [46] (Multimedia Appendix 9). At follow-up, self-reported PA effects remained unchanged (Multimedia Appendix 10). In sensitivity analyses omitting outliers, objective and self-reported PA results were stable, but step count effects became significant after removing outliers or high-risk-of-bias studies (SMD=0.33, 95% CI 0.04-0.61; *P*=.02; *I*²=0%). No outliers were identified at follow-up, and results were consistent when excluding high-risk-of-bias studies.

#### Publication Bias

We observed asymmetric funnel plots for all outcomes in this study (Multimedia Appendix 11), suggesting potential publication bias. However, the Egger tests indicated no significant publication bias for objective PA (*P*=.35), self-reported PA (*P*=.41), or step count (*P*=.60) at postintervention, or self-reported PA at follow-up (*P*=.07), suggesting an absence of small-study effects.

## Discussion

### Principal Results

To our knowledge, this study is the first to synthesize the effects of mobile phone messaging–based interventions to promote PA among patients with cancer. The pooled results showed that mobile phone messaging–based intervention is effective for increasing objective PA levels at postintervention, but not for self-reported PA levels or step count. No significant effect was observed on longer-term PA improvement. Subgroup analyses suggested that targeting post-treatment patients with cancer and adopting more BCTs significantly affected intervention effects.

### Comparison With Prior Work

A statistically significant yet modest effect of mobile phone messaging–based interventions on promoting objective PA levels was identified. Notably, previous reviews of messaging interventions among adult populations revealed a relatively smaller effect in improving objective PA level (Hedge g=0.31) [25]. One of the possible explanations may be related to the use of theory. Four out of 5 (80%) RCTs measuring objective PA in this review were driven by theories [30,41,46,48], while only 1 out of 5 studies in the previous review had a theoretical basis [49]. Besides, the previous review targeted a wide range of adult populations, including those with noncommunicable diseases and those without [25]. Individuals with varying health conditions may have different needs for PA promotion, and therefore, the effect of mobile messaging may be obscured. Furthermore, the effect of mobile phone messaging on objective PA promotion as revealed by our study is also relatively larger than eHealth interventions conducted among patients with cancer (SMD=0.19) [15]. This may be explained by the special features of mobile phone messaging, such as low cost, convenience, and high accessibility to real-time reminders and support [19, 50]. Future messaging interventions and eHealth trials should explore how the interventions work, such as by conducting process evaluation and mediation analyses.

Despite the significant effect of mobile phone messaging–based interventions on objective PA, no significant effect on self-reported PA levels or step count was identified. For self-reported PA, it could be affected by information bias (ie, varied understanding of PA estimates) related to individual intelligence and educational level [51] and recall bias [52]. Therefore, self-reported PA measures have been regarded as less valid PA measurement compared to objective measures [53]. For step count, it includes estimates of PA during periods of activity, leisure activity, lower body movements, and sporadic movements in daily life and does not estimate some PA, such as biking and swimming [54]. Therefore, step count provides data on the total volume of ambulatory activity regardless of intensity and is different from the objective PA estimates, which are mostly time-based estimates of PA of particular intensities. These may account for the differences in the effect of mobile phone messaging–based interventions on objective PA and step count.

The results of subgroup analyses revealed 2 factors associated with the effect of mobile messaging on promoting step count. The first factor was targeting patients after cancer treatment. The physical and psychological demands of active cancer treatment can make it challenging for patients to maintain or increase PA levels [55], so PA promotion targeting post-treatment patients is more likely to succeed. The other factor was related to the use of more BCTs. Mobile messaging allows for enactment of a wide range of BCTs through real-time and tailored communication, which may enhance individuals’ engagement in and adherence to PA [19,56]. For BCTs, the included trials adopted a median of 10 (IQR 8.5-13.5) BCTs, with the most frequently used ones being self-monitoring of behavior, instructions on how to perform behaviors, habit formation, and nonspecific reward. With the use of more BCTs, interventions may be able to systematically address key factors that influence behavior change, such as goal-setting, self-efficacy, and self-monitoring [57,58]. Despite the importance of BCTs in behavioral change studies, only 2 of the 13 (15%) included studies explicitly reported the adoption of BCTs [37,46]. Future studies should consider improving the reporting of message development based on the adoption of BCTs and targeting patients who have finished active treatment. Also, more research is needed to determine the optimal combination of theories, BCTs, and intervention regimens (eg, timing and frequency) of messaging to influence PA behaviors.

The sensitivity analyses demonstrated that the overall results were relatively robust. The significant effect of mobile phone messaging–based interventions on objective PA and the nonsignificant effect on self-reported PA persisted after the exclusion of obvious outliers and studies with a high risk of bias. However, the effect on step count, which was nonsignificant in the primary analysis, became significant following the removal of the studies by Kenfield et al [46] and Villaron et al [39]. This shift may be attributed to the high risk of bias in these studies, which potentially introduced distortion into the pooled estimate. Furthermore, leave-one-out sensitivity analysis revealed that the significant effect on objective PA was contingent upon the inclusion of two influential studies: Gell et al [30] and Kenfield et al [46]. The instability observed upon their removal can be explained by their distinct characteristics. The study by Kenfield et al [46] was judged to be at high risk of bias, which may have biased the overall result. In contrast, the study by Gell et al [30], while methodologically sounder, presented an exceptionally large effect size and carried substantial weight in the meta-analysis due to its sample size. More methodologically rigorous trials should be conducted to test the effects of mobile phone messaging-based interventions on PA promotion.

### Limitations

This review has some limitations. First, the geographical scope of the studies in this review was confined to Western and high-income countries, so the generalizability of the findings could be restricted. Second, despite comprehensive efforts, the literature search may have missed potentially relevant papers. Third, the nature of mobile phone messaging–based interventions made it hard to blind participants, which may have biased the effects of the interventions. Fourth, the subgroup analyses were exploratory, with no formal tests for interaction due to the limited number of studies; this approach is prone to ecological fallacy and overinterpretation. In addition, substantial heterogeneity in some subgroups and the small number of studies further hindered the robust interpretation of those findings. Fifth, only 2 studies estimated PA levels at follow-up, leading to unclear long-term effects of mobile phone messaging–based interventions. Finally, although our search included major English and Chinese databases to capture a broad evidence base, the exclusion of studies in other languages (eg, Spanish and Portuguese) introduced a potential for language bias.

### Implications

Mobile phone messaging–based interventions could be recommended to improve PA among patients with cancer, given their significant effect on objective PA, an outcome that is generally considered to be more valid than self-reported PA and step count [53]. Health care providers should improve their capacity for designing and implementing mobile phone messaging–based interventions on PA promotion. Mobile phone messaging–based interventions that are designed based on theories, adopt various BCTs, and target patients who have finished active treatment are more likely to elicit significant benefits on diverse PA estimates. Importantly, mobile phone messaging–based interventions should be implemented among patients who have no contraindications to unsupervised exercise. Education on safety precautions for home-based exercise should be provided to avoid potential exercise-related AEs.

In terms of research implications, future studies should prioritize more methodologically rigorous RCTs to strengthen the evidence base for mobile phone messaging–based interventions, given that only 30.8% of the included trials were at low risk of bias. Development processes for messages in the intervention should also be rigorous and clearly reported by specifying the theoretical basis and BCTs adopted. Furthermore, studies focusing on exploring differential intervention regimens (eg, message frequency and duration, and wearable devices) should be conducted to promote understanding of the optimal messaging intervention design. Besides, longer follow-up should be considered for future research to estimate the long-term effects of mobile phone messaging–based interventions. Finally, instant messaging is increasingly used in other messaging interventions on behavior change, such as smoking cessation [59]; however, it is not adopted by any of the included studies in this review. Future messaging interventions for promoting PA in patients with cancer can adopt instant messaging as the message delivery platform because it is free of charge, routinely used in daily life, and has a broad range of functionalities (eg, sharing large files in various media formats, such as images, videos, documents, and voice messages).

### Conclusions

Our findings indicate that the effect of mobile phone messaging–based interventions varies among different PA outcomes. While messaging significantly improved objective PA with a small effect size, its effect on self-reported PA and step count was insignificant. Mobile phone messaging–based interventions adopting more BCTs and targeting patients who have completed active cancer treatment were more likely to improve step count. More methodologically rigorous trials are needed to test the long-term effect of mobile phone messaging–based intervention on PA and to explore the effects of different intervention regimens.

## Supplementary material

10.2196/73934 Multimedia Appendix 1 Search strategies.

10.2196/73934 Multimedia Appendix 2 Behavior change techniques (BCTs) used in each study.

10.2196/73934 Multimedia Appendix 3 Subgroups of the included trials.

10.2196/73934 Multimedia Appendix 4 Outcome measurement for each study.

10.2196/73934 Multimedia Appendix 5 Grade of recommendation, assessment, development, and evaluation (GRADE) assessment.

10.2196/73934 Multimedia Appendix 6 Subgroup analysis of treatment status and number of behavior change techniques (BCTs) for step count.

10.2196/73934 Multimedia Appendix 7 Sensitivity analysis for self-reported PA at post-intervention.

10.2196/73934 Multimedia Appendix 8 Sensitivity analysis for step count at postintervention.

10.2196/73934 Multimedia Appendix 9 Sensitivity analysis for objective physical activity (PA) levels at postintervention.

10.2196/73934 Multimedia Appendix 10 Sensitivity analysis for self-reported physical activity (PA) levels at follow-up.

10.2196/73934 Multimedia Appendix 11 Funnel plots.

10.2196/73934 Checklist 1 PRISMA checklist.

## Abbreviations

AE: adverse event
BCT: behavior change technique
GRADE: grade of recommendation, assessment, development, and evaluation
HIPAA: Health Information Portability and Accountability Act
MVPA: moderate-to-vigorous physical activity
PA: physical activity
PRISMA: Preferred Reporting Items for Systematic Reviews and Meta-Analyses
RCT: randomized controlled trial
SMD: standardized mean difference
