# Supplementary material for: Mobile Phone Messaging–Based Interventions to Improve Physical Activity in Patients With Cancer: Systematic Review and Meta-Analysis
Source: J Med Internet Res. 2025 Dec 15;27:e73934. doi: 10.2196/73934 (PMC12704914; doi:10.2196/73934)
Supplement: Multimedia Appendix 1 [file jmir-v27-e73934-s001.docx]

**Multimedia Appendix 1. Search strategies**

Pubmed

#1 (Neoplasms [Mesh]) OR ((cancer[Title/Abstract]) OR (malignan*[Title/Abstract]) OR (oncolog*[Title/Abstract]) OR (tumour[Title/Abstract]) OR (tumor[Title/Abstract]) OR (carcinoma*[Title/Abstract]) OR (leukemia[Title/Abstract]) OR (leukaemia[Title/Abstract]) OR (sarcoma*[Title/Abstract])) OR ((Cancer survivors [Title/Abstract]) OR (Cancer patients [Title/Abstract]) OR (Patients with cancer [Title/Abstract]))

#2 (telemedicine [Mesh]) OR (Mobile phone messaging [Title/Abstract]) OR (Text messag* [Mesh]) OR (Messag* [Title/Abstract]) OR (Texting [Title/Abstract]) OR (telephone [Title/Abstract]) OR (Mobile Phone [Title/Abstract]) OR (Telehealth [Title/Abstract]) OR (smartphone [Mesh]) OR (smart phone [Title/Abstract]))

#3 ((Exercise [Mesh]) OR (Sports [Mesh]) OR (Physical Exertion [Mesh]) OR (Motor Activity [Mesh])) OR (Physical activ*[Title/Abstract]) OR (Physical fitness [Title/Abstract]) OR (aerobic capacity [Title/Abstract]) OR (strength training [Title/Abstract]) OR (resistance training [Title/Abstract]) OR (functional training [Title/Abstract]))

#4 ((Randomized Controlled Trial [Mesh]) OR (Clinical Trial [Mesh])) OR ((single blind procedure [Title/Abstract]) OR (double blind [Title/Abstract]) OR (trials [Title/Abstract]) OR (placebo [Title/Abstract])

#5 #1 AND #2 AND #3 (Filters: Randomized Controlled Trial)

#6 #1 AND #2 AND #3 AND #4

Web of Science

TS=( Neoplasms OR cancer OR malignan* OR oncolog* OR tumour OR tumor OR carcinoma* OR leukemia OR leukaemia OR sarcoma OR "cancer survivors" OR "cancer patients" OR "patients with cancer") AND TS=("mobile phone messaging" OR "text messag*" OR messag* OR Texting OR telemedicine OR smartphone OR "smart phone" OR "mobile application") AND TS=( exercise OR sports OR exercise* OR "physical active*" OR "physical fitness" OR "aerobic capacity" OR "strength training" OR "resistance training" OR "functional training") AND TS=( "randomized controlled trial" OR "clinical trial" OR "single blind procedure" OR "double blind" OR trials OR "placebo") NOT TI=(protocol OR review))

MEDLINE

#1 (Neoplasms or cancer or malignan* or oncolog* or tumour or tumor or carcinoma* or leukemia or leukaemia or sarcoma or "cancer survivors" or "cancer patients" or "patients with cancer").ab,ti,kw.

#2 (telemedicine or "mobile phone messaging" or "text messag*" or messag* or Texting or telephone or telehealth or "mobile health" or smartphone or "smart phone").ab,ti,kw.

#3 (exercise or sports or "physical exertion" or "motor activity" or "physical active*" or "physical fitness" or "aerobic capacity" or "strength training" or "resistance training" or "functional training").ab,ti,kw.

#4 ("randomized controlled trial" or "clinical trial" or "single blind" or "double blind" or trials or placebo).ab,ti,kw.

#5 1 and 2 and 3 and 4

#6 1 and 2 and 3

#7 limit 5 to (clinical study or randomized controlled trial or randomized controlled trial)

#8 limit 6 to (clinical study or randomized controlled trial or randomized controlled trial)

EMBASE

#1 (Neoplasms or cancer or malignan* or oncolog* or tumour or tumor or carcinoma* or leukemia or leukaemia or sarcoma or "cancer survivors" or "cancer patients" or "patients with cancer").ab,ti,kw.

#2 (telemedicine or "mobile phone messaging" or "text messag*" or messag* or Texting or telephone or “mobile phone” or "mobile health" or smartphone or "smart phone").ab,ti,kw.

#3 (exercise or sports or "physical exertion" or "motor activity" or "physical active*" or "physical fitness" or "aerobic capacity" or "strength training" or "resistance training" or "functional training").ab,ti,kw.

#4 ("randomized controlled trial" or "clinical trial" or "single blind" or "double blind" or trials or RCT).ab,ti,kw.

#5 1 and 2 and 3 and 4

#6 1 and 2 and 3

#7 limit 5 to (clinical study or randomized controlled trial or randomized controlled trial) and "remove medline records")

#8 limit 6 to (clinical study or randomized controlled trial or randomized controlled trial) and "remove medline records")

#9 limit 5 to ("remove clinical trial (clinicaltrials.gov) records" and "remove medline records")

#10 limit 6 to ("remove clinical trial (clinicaltrials.gov) records" and "remove medline records")

Scopus

( TITLE-ABS-KEY ( Neoplasms OR cancer OR malignan* OR oncolog* OR tumour OR tumor OR carcinoma* OR leukemia OR leukaemia OR sarcoma OR "cancer survivors" OR "cancer patients" OR "patients with cancer" ) AND TITLE-ABS-KEY ( telemedicine OR "mobile phone messaging" OR "text messag*" OR messag* OR Texting OR telephone OR telehealth OR "mobile health" OR smartphone OR "smart phone" ) AND TITLE-ABS-KEY ( exercise OR sports OR exercise* OR "physical active*" OR "physical fitness" OR "aerobic capacity" OR "strength training" OR "resistance training" OR "functional training" ) AND TITLE-ABS-KEY ( "randomized controlled trial" OR "clinical trial" OR "single blind" OR "double blind" OR trials ) AND NOT TITLE ( "protocol" OR "review") )

( TITLE-ABS-KEY ( Neoplasms OR cancer OR malignan* OR oncolog* OR tumour OR tumor OR carcinoma* OR leukemia OR leukaemia OR sarcoma OR "cancer survivors" OR "cancer patients" OR "patients with cancer" ) AND TITLE-ABS-KEY ( telemedicine OR "mobile phone messaging" OR "text messag*" OR messag* OR Texting OR telephone OR telehealth OR "mobile health" OR smartphone OR "smart phone" ) AND TITLE-ABS-KEY ( exercise OR sports OR exercise* OR "physical active*" OR "physical fitness" OR "aerobic capacity" OR "strength training" OR "resistance training" OR "functional training" ) AND TITLE-ABS-KEY ( "randomized controlled trial" OR "clinical trial" OR "single blind" OR "double blind" OR trials ) AND NOT TITLE ( "protocol" OR "review" ) ) AND ( LIMIT-TO ( DOCTYPE , "ar" ) ) AND ( LIMIT-TO ( LANGUAGE , "English" ) OR LIMIT-TO ( LANGUAGE , "Chinese" ) ) AND ( LIMIT-TO ( SRCTYPE , "j" ) ) AND ( LIMIT-TO ( OA , "all" ) )

Cochrane Library

(Neoplasms OR cancer OR malignan* OR oncolog* OR tumour OR tumor OR carcinoma* OR leukemia OR leukaemia OR sarcoma OR "cancer survivors" OR "cancer patients" OR "patients with cancer"):ti,ab,kw AND (telemedicine OR "mobile phone messaging" OR (text NEXT messag*) OR messag* OR Texting OR telephone OR telehealth OR “mobile health” OR smartphone OR “smart phone”):ti,ab,kw AND (exercise OR sports OR "physical exertion" OR "motor activity" OR (physical NEXT active*) OR "physical fitness" OR "aerobic capacity" OR "strength training" OR "resistance training" OR "functional training"):ti,ab,kw AND ((randomized OR randomised OR randomization OR randomisation OR placebo OR "randomly allocated" OR "allocated randomly" OR trial OR groups)):ti,ab,kw

(Neoplasms OR cancer OR malignan* OR oncolog* OR tumour OR tumor OR carcinoma* OR leukemia OR leukaemia OR sarcoma OR "cancer survivors" OR "cancer patients" OR "patients with cancer"):ti,ab,kw AND (telemedicine OR "mobile phone messaging" OR (text NEXT messag*) OR messag* OR Texting OR telephone OR telehealth OR “mobile health” OR smartphone OR “smart phone”):ti,ab,kw AND (exercise OR sports OR "physical exertion" OR "motor activity" OR (physical NEXT active*) OR "physical fitness" OR "aerobic capacity" OR "strength training" OR "resistance training" OR "functional training"):ti,ab,kw AND ((randomized OR randomised OR randomization OR randomisation OR placebo OR "randomly allocated" OR "allocated randomly" OR trial OR groups)):ti,ab,kw NOT (protocol).ti,kw.

Wanfang

(主题:(癌症 或 肿瘤 或 癌症患者 或 恶性肿瘤 或 肿瘤患者 或 癌 或 瘤)) 与 (主题:(短信 或 即时通信 或 移动电话 或 微信 或 手机 或 APP 或 智能手机 或 移动健康 或 移动医疗)) 与 (主题:(运动 或 体能运动 或 体育活动 或 身体活动 或 体能活动 或 运动锻炼 或 体育锻炼)) 与 (主题:(随机对照 或 随机 或 RCT 或 试验 或 随机分组 或 随机分配))

Chinese National Knowledge Infrastructure

((主题：癌症)OR(主题：肿瘤)OR(主题：癌症患者)OR(主题：恶性肿瘤)OR(主题：肿瘤患者)OR(主题：癌)OR(主题：瘤))AND((主题：短信)OR(主题：即時通信)OR (主题：移动电话)OR(主题：微信)OR(主题：手机)OR(主题：APP) OR (主题：智能手机))AND((主题：运动)OR(主题：体能运动)OR(主题：体育活动)OR(主题：身体活动)OR(主题：体能活动)OR(主题：运动锻炼))AND((主题：随机对照)OR(主题：随机对照)OR(主题：随机)OR(主题：RCT)OR(主题：随机对照)OR(主题：试验)

ProQuest Dissertations & Theses

(Neoplasms OR cancer OR malignan* OR oncolog* OR tumour OR tumor OR carcinoma* OR leukemia OR leukaemia OR sarcoma OR "cancer survivors" OR "cancer patients" OR "patients with cancer" ) AND ( telemedicine OR "mobile phone messaging" OR "text messag*" OR messag* OR Texting OR telephone OR telehealth OR "mobile health" OR smartphone OR "smart phone" ) AND ( exercise OR sports OR exercise* OR "physical active*" OR "physical fitness" OR "aerobic capacity" OR "strength training" OR "resistance training" OR "functional training" ) AND ( "randomized controlled trial" OR "clinical trial" OR "single blind" OR "double blind" OR trials )

Electronic Theses and Dissertations (EBSCO Open Dissertations)

(Neoplasms OR cancer OR malignan* OR oncolog* OR tumour OR tumor OR carcinoma* OR leukemia OR leukaemia OR sarcoma OR "cancer survivors" OR "cancer patients" OR "patients with cancer") AND (telemedicine OR "mobile phone messaging" OR "text messag*" OR messag* OR Texting OR telephone OR telehealth OR "mobile health" OR smartphone OR "smart phone") AND (exercise OR sports OR exercise* OR "physical active*" OR "physical fitness" OR "aerobic capacity" OR "strength training" OR "resistance training" OR "functional training") AND ("randomized controlled trial" OR "clinical trial" OR "single blind" OR "double blind" OR trials)

OpenGrey

(Neoplasms OR cancer OR malignan* OR oncolog* OR tumour OR tumor OR carcinoma* OR leukemia OR leukaemia OR sarcoma OR "cancer survivors" OR "cancer patients" OR "patients with cancer") AND (telemedicine OR "mobile phone messaging" OR "text messag*" OR messag* OR Texting OR telephone OR telehealth OR "mobile health" OR smartphone OR "smart phone") AND (exercise OR sports OR exercise* OR "physical active*" OR "physical fitness" OR "aerobic capacity" OR "strength training" OR "resistance training" OR "functional training") AND ("randomized controlled trial" OR "clinical trial" OR "single blind" OR "double blind" OR trials)
