# Supplementary material for: Mobile Phone Messaging–Based Interventions to Improve Physical Activity in Patients With Cancer: Systematic Review and Meta-Analysis
Source: J Med Internet Res. 2025 Dec 15;27:e73934. doi: 10.2196/73934 (PMC12704914; doi:10.2196/73934)
Supplement: Multimedia Appendix 2 [file jmir-v27-e73934-s002.docx]

# Multimedia Appendix 2

*BCTs used in each study*

| BCTs | Allicock et al. 2021 | Bade et al. 2021 | Gell et al. 2020 | Gomersall et al. 2019 | Haggerty et al. 2019 | Kenfield et al. 2019 | Singletion et al. 2023 | Van blarigan et al. 2019 | Villaron et al. 2018 | Walsh et al. 2021 | SenthilKumar et al. 2024 | Chan et al. 2020 | Hassoon et al. 2021 |
| --- | --- | --- | --- | --- | --- | --- | --- | --- | --- | --- | --- | --- | --- |
| Goal setting (behavior) |  | √ | √ |  | √ | √ |  |  |  | √ | √ | √ | √ |
| Problem solving |  |  | √ | √ |  | √ |  |  |  | √ |  |  |  |
| Goal setting (outcome) |  |  |  | √ |  |  |  | √ |  | √ | √ | √ | √ |
| Action planning |  |  | √ | √ | √ | √ |  | √ |  | √ | √ | √ | √ |
| Review behavior goal(s) |  |  | √ |  |  |  |  |  |  | √ |  |  | √ |
| Discrepancy between  current behavior and  goal |  | √ | √ | √ |  |  |  |  |  |  |  |  |  |
| Review outcome goal(s) |  |  |  | √ |  |  |  |  |  |  |  |  |  |
| Feedback on behavior |  | √ | √ |  | √ |  |  |  |  | √ |  | √ | √ |
| Self-monitoring of  behavior | √ |  |  |  |  | √ |  |  | √ | √ | √ |  | √ |
| Self-monitoring of  outcome(s) of behavior | √ | √ | √ | √ |  | √ | √ | √ | √ | √ | √ | √ | √ |
| Feedback on outcome(s)  of behavior |  |  |  | √ |  |  |  |  |  |  |  |  | √ |
| Social support  (unspecified) | √ |  | √ | √ | √ | √ | √ |  | √ |  | √ | √ |  |
| Social support  (emotional) |  |  |  |  |  |  | √ |  |  |  | √ | √ |  |
| Instruction on how to  perform a behavior | √ |  | √ | √ |  |  |  |  |  | √ | √ |  |  |
| information about  health consequences | √ | √ | √ | √ | √ | √ | √ | √ |  | √ | √ |  |  |
| Information about social  and environmental  consequence |  |  |  |  |  |  | √ |  |  |  |  |  |  |
| Monitoring of emotional  consequences |  |  |  |  |  |  |  |  | √ |  |  |  |  |
| Information about  emotional consequences |  |  |  |  |  |  | √ |  |  |  |  |  |  |
| Demonstration of the behavior |  |  |  | √ |  | √ |  |  |  | √ |  |  |  |
| Prompts/cues | √ | √ | √ | √ | √ | √ |  | √ |  |  | √ | √ |  |
| Behavioral practice |  |  |  |  |  | √ |  |  |  |  |  |  |  |
| Behavior substitution |  |  |  | √ |  | √ |  |  |  |  |  |  |  |
| Habit formation |  | √ | √ | √ | √ | √ |  | √ |  | √ | √ | √ | √ |
| Habit reversal |  |  |  | √ |  |  |  |  |  |  |  |  |  |
| Graded tasks |  |  |  |  |  |  |  |  |  | √ |  |  |  |
| Credible source |  |  |  |  |  | √ |  |  |  |  |  |  |  |
| Pros and cons |  |  |  |  |  |  |  | √ |  |  |  |  |  |
| Comparative imagining of future outcomes |  |  |  |  |  |  |  |  |  |  |  |  |  |
| Material reward (behavior) | √ | √ |  |  |  |  |  | √ |  |  |  |  |  |
| Non-specific reward | √ | √ | √ | √ | √ | √ | √ | √ | √ | √ | √ | √ | √ |
| Adding objects to the environment |  |  |  |  |  | √ |  |  |  |  |  |  |  |
| Valued self-identify |  |  |  |  |  | √ |  |  |  |  |  |  |  |
| Verbal persuasion about capability | √ |  |  |  |  |  |  |  |  |  |  |  |  |
| Total no. of BCTS | 9 | 9 | 13 | 16 | 8 | 16 | 7 | 9 | 5 | 14 | 12 | 10 | 10 |

From: Michie S, Richardson M, Johnston M, Abraham C, Francis J, Hardeman W, et al. The behavior change technique taxonomy (v1) of 93 hierarchically clustered techniques: building an international consensus for the reporting of behavior change interventions. Ann Behav Med. 2013 Aug;46(1):81-95.
