# Supplementary material for: Mobile Phone Messaging–Based Interventions to Improve Physical Activity in Patients With Cancer: Systematic Review and Meta-Analysis
Source: J Med Internet Res. 2025 Dec 15;27:e73934. doi: 10.2196/73934 (PMC12704914; doi:10.2196/73934)
Supplement: Multimedia Appendix 3 [file jmir-v27-e73934-s003.docx]

# Multimedia Appendix 3

*Subgroups of the Included Trials*

| Study | Treatment status | Cancer types | Intervention period | Intervention frequency | Interactive message | Tailored message | Wearable device | No. of BCTs | Theory basis |
| --- | --- | --- | --- | --- | --- | --- | --- | --- | --- |
| Allicock et al. 2021 | Post treatment | Single cancer | ≤3 months | Daily | No | Yes | No | <10 | Yes |
| Bade et al. 2021 | Under treatment | Single cancer | ≤3 months | Daily | No | No | Yes | <10 | No |
| Gell et al. 2020 | Post treatment | Mixed cancer | ≤3 months | Less than daily | No | Yes | Yes | ≥10 | Yes |
| Gomersall et al. 2019 | Post treatment | Mixed cancer | ≤3 months | Less than daily | Yes | Yes | No | ≥10 | No |
| Haggerty et al. 2017 | Post treatment | Single cancer | > 3 months | Daily | Yes | Yes | No | <10 | No |
| Kenfield et al. 2019 | Post treatment | Single cancer | ≤3 months | Less than daily | Yes | No | Yes | ≥10 | Yes |
| Singleton et al. 2023 | Post treatment | Single cancer | > 3 months | Less than daily | No | No | No | <10 | No |
| Van Blarigan et al. 2019 | Post treatment | Single cancer | ≤3 months | Daily | No | No | Yes | <10 | Yes |
| Villaron et al. 2018 | Under treatment | Mixed cancer | ≤3 months | Less than daily | No | No | Yes | <10 | No |
| Walsh et al. 2021 | Post treatment | Mixed cancer | ≤3 months | Less than daily | No | Yes | Yes | ≥10 | No |
| SenthilKumar et al. 2024 | Scheduled treatment | Single cancer | ≤3 months | Less than daily | No | No | Yes | ≥10 | Yes |
| Chan et al. 2020 | Not limited | Single cancer | ≤3 months | Less than daily | No | No | Yes | ≥10 | Yes |
| Hassoon et al. 2021 | Post treatment | Mixed cancer | ≤3 months | Daily | No | Yes | Yes | ≥10 | Yes |
