# Supplementary material for: Mobile Phone Messaging–Based Interventions to Improve Physical Activity in Patients With Cancer: Systematic Review and Meta-Analysis
Source: J Med Internet Res. 2025 Dec 15;27:e73934. doi: 10.2196/73934 (PMC12704914; doi:10.2196/73934)
Supplement: Multimedia Appendix 4 [file jmir-v27-e73934-s004.docx]

Multimedia Appendix 4. Studies contributing to each outcome

| Outcomes | Parameters | Studies |
| --- | --- | --- |
| Objective PA | MVPA min/week | Gell et al. 2020 |
|  | MVPA min/16 h awake | Gomersall et al. 2019 |
|  | MVPA min/day | Van Blarigan et al. 2019; Kenfield et al. 2019 |
|  | Total PA/VM | Allicock et al. 2021 |
| Self-reported PA | MVPA (BRFSS, MAD, MARCA, self-administered questionnaire, CHAMPS) | Allicock et al. 2021, Bade et al. 2021; Gomersall et al. 2019; Kenfield et al. 2019; Walsh et al. 2021 |
|  | Total PA (IPAQ, GPAQ, GLTEQ, GLPA) | Haggerty et al. 2017; Singleton et al. 2023; Walsh et al. 2021; SenthilKumar et al. 2024 |
| Step count | Step count (Fitbit) | Kenfield et al. 2019; van Blarigan et al. 2019; Walsh et al. 2021; Hassoon et al. 2021 |
|  | Step count (pedometer) | Villaron et al. 2018 |

Notes: VM, vector magnitude; BRFSS, the Behavioral Risk Factor Surveillance System; MAD, the modified Activity Questionnaire); MARCA, the Multimedia Activity Recall for Children and Adults; CHAMPS, Community Health Activities Model Program for Seniors Survey; IPAQ, the International Physical Activity Questionnaire Short Form; GPAQ, the Global Physical Activity Questionnaire; GLTEQ, Godin Leisure-Time Exercise Questionnaire; GLPA, Godin Leisure Physical Activity
