# Supplementary material for: Mobile Phone Messaging–Based Interventions to Improve Physical Activity in Patients With Cancer: Systematic Review and Meta-Analysis
Source: J Med Internet Res. 2025 Dec 15;27:e73934. doi: 10.2196/73934 (PMC12704914; doi:10.2196/73934)
Supplement: Multimedia Appendix 5 [file jmir-v27-e73934-s005.docx]

Multimedia Appendix 5. GRADE assessment

| **N**o. of studies | Study design | Risk of Bias | Inconsistency | Indirectness | Imprecision | Publication bias | Certainty |
| --- | --- | --- | --- | --- | --- | --- | --- |
| **Objective PA at post-intervention** | | | | | | | |
| 5 | randomised trials | serious | not serious | not serious | not serious | none | ⨁⨁⨁⨁ High |
| **Self-reported PA at post-intervention** | | | | | | | |
| **9** | randomised trials | very serious | not serious | not serious | not serious | none | ⨁◯◯◯ Very low |
| **Step count at post-intervention** | | | | | | | |
| 5 | randomised trials | serious | not serious | not serious | not serious | none | ⨁⨁⨁◯ Moderate |
| **Self-reported PA at follow-up** | | | | | | | |
| 4 | randomised trials | not serious | not serious | not serious | not serious | none | ⨁⨁⨁◯ Moderate |
