# Supplementary figures and images for: Mobile Phone Messaging–Based Interventions to Improve Physical Activity in Patients With Cancer: Systematic Review and Meta-Analysis
Source: J Med Internet Res. 2025 Dec 15;27:e73934. doi: 10.2196/73934 (PMC12704914; doi:10.2196/73934)

# Multimedia Appendix 6

*Subgroup analysis of treatment status and number of BCTs for step count*


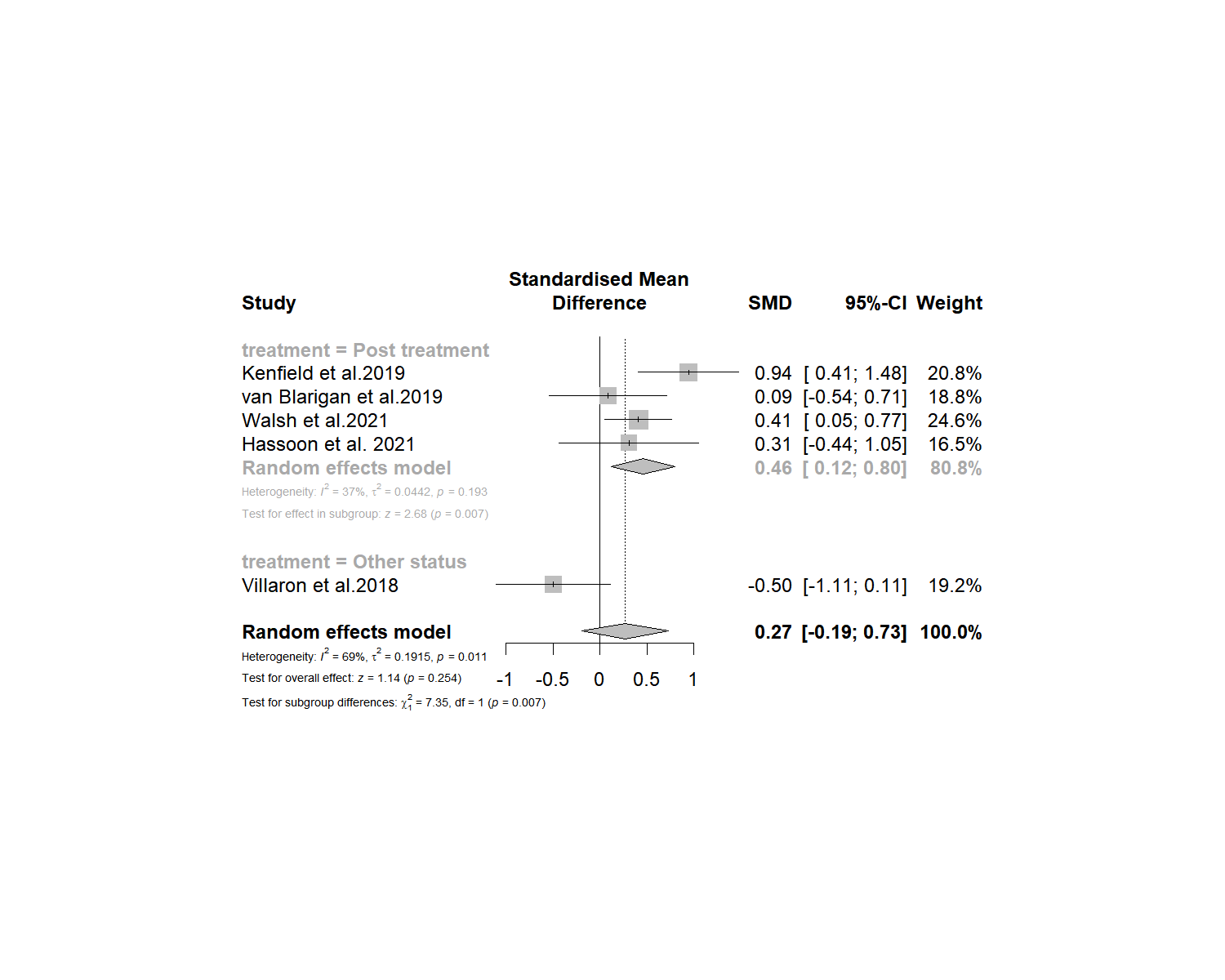


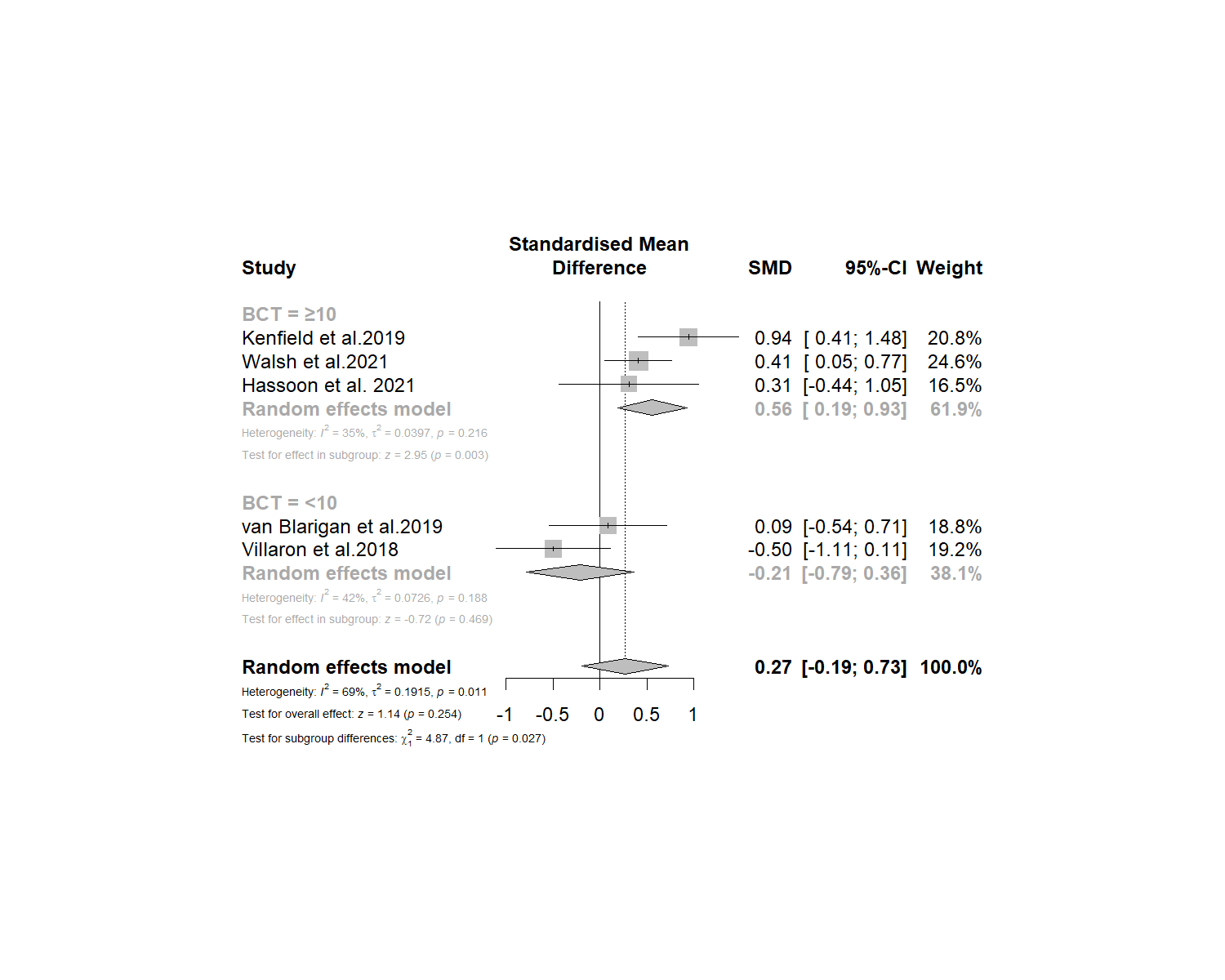

Supplement: Multimedia Appendix 6 [file jmir-v27-e73934-s006.docx]

# Multimedia Appendix 10

*Sensitivity Analysis for self-reported PA levels at follow-up*


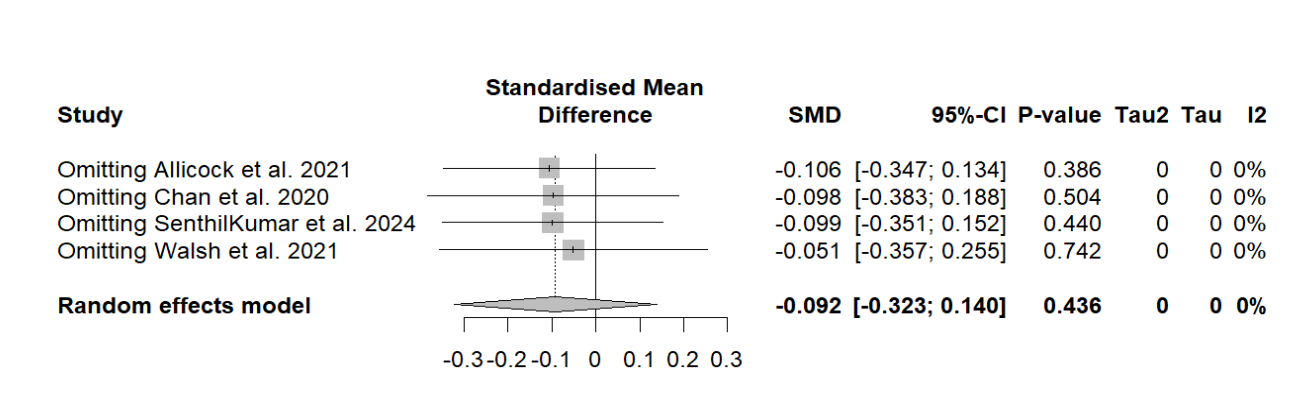

Supplement: Multimedia Appendix 10 [file jmir-v27-e73934-s010.docx]
