# Supplementary material for: Mobile Phone Messaging–Based Interventions to Improve Physical Activity in Patients With Cancer: Systematic Review and Meta-Analysis
Source: J Med Internet Res. 2025 Dec 15;27:e73934. doi: 10.2196/73934 (PMC12704914; doi:10.2196/73934)

# Multimedia Appendix 9

*Sensitivity analysis for objective PA levels at post-intervention (leave-one-out)*


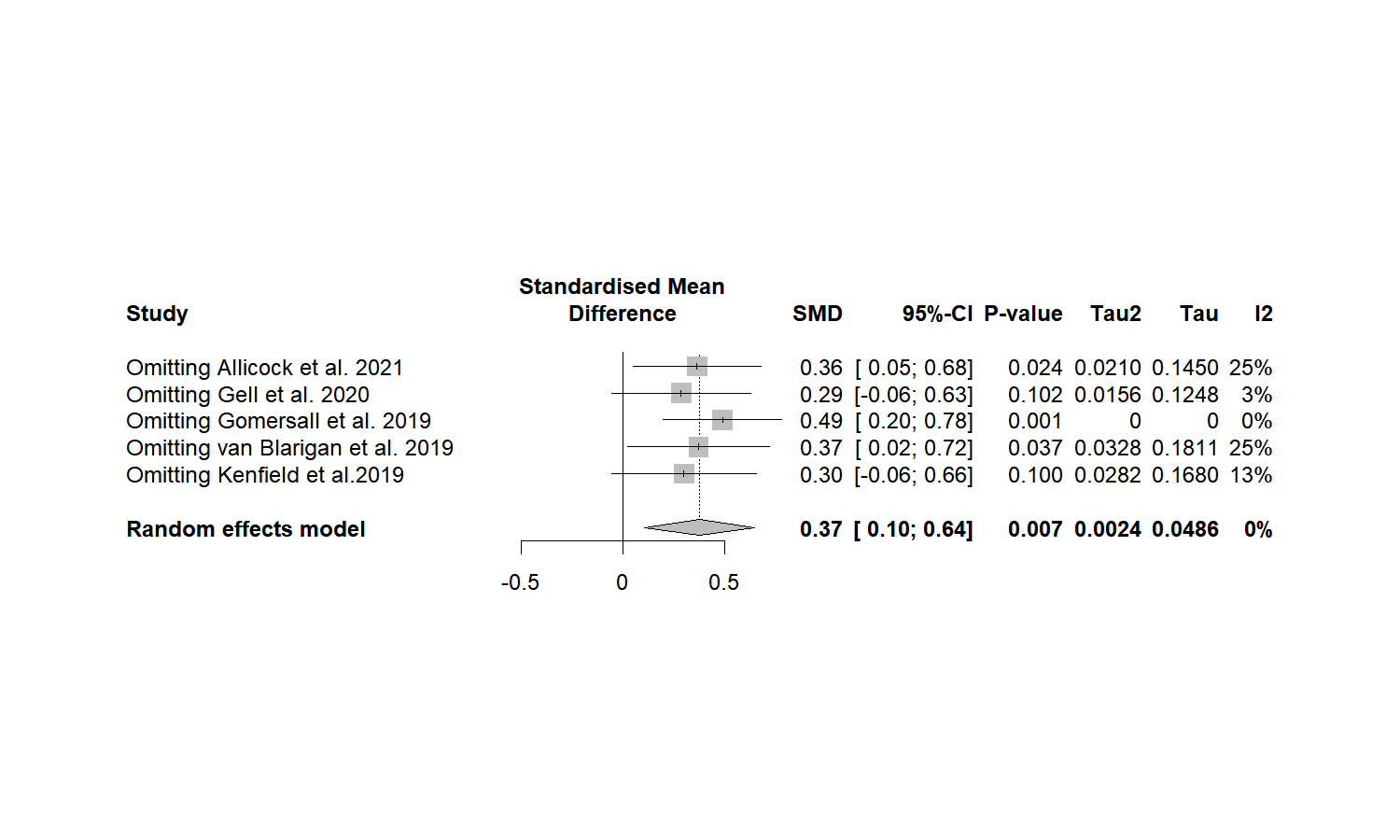


*Sensitivity analysis for objective PA levels at post-intervention (without outliers)*


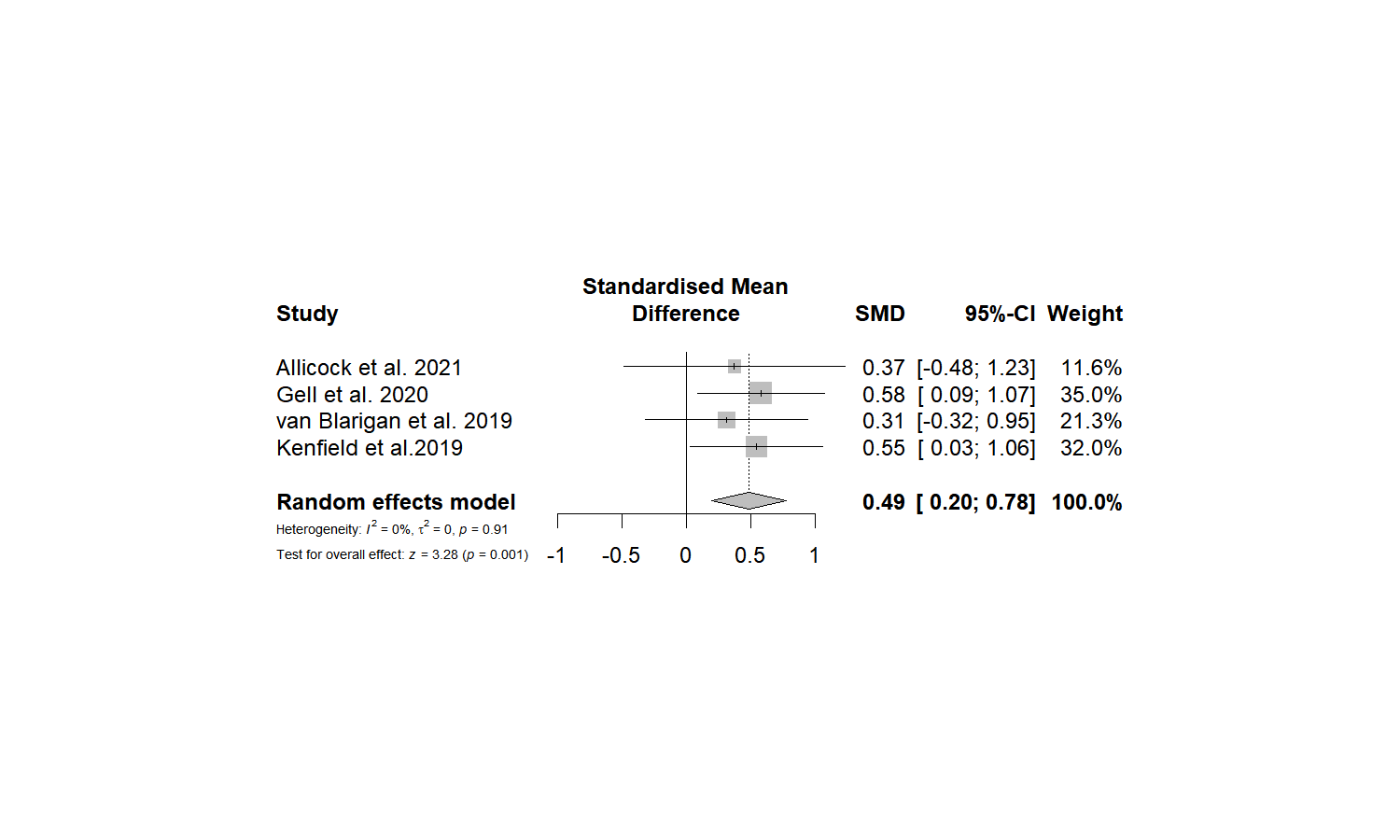


*Sensitivity analysis for objective PA levels at post-intervention (without studies with high risk of bias)*


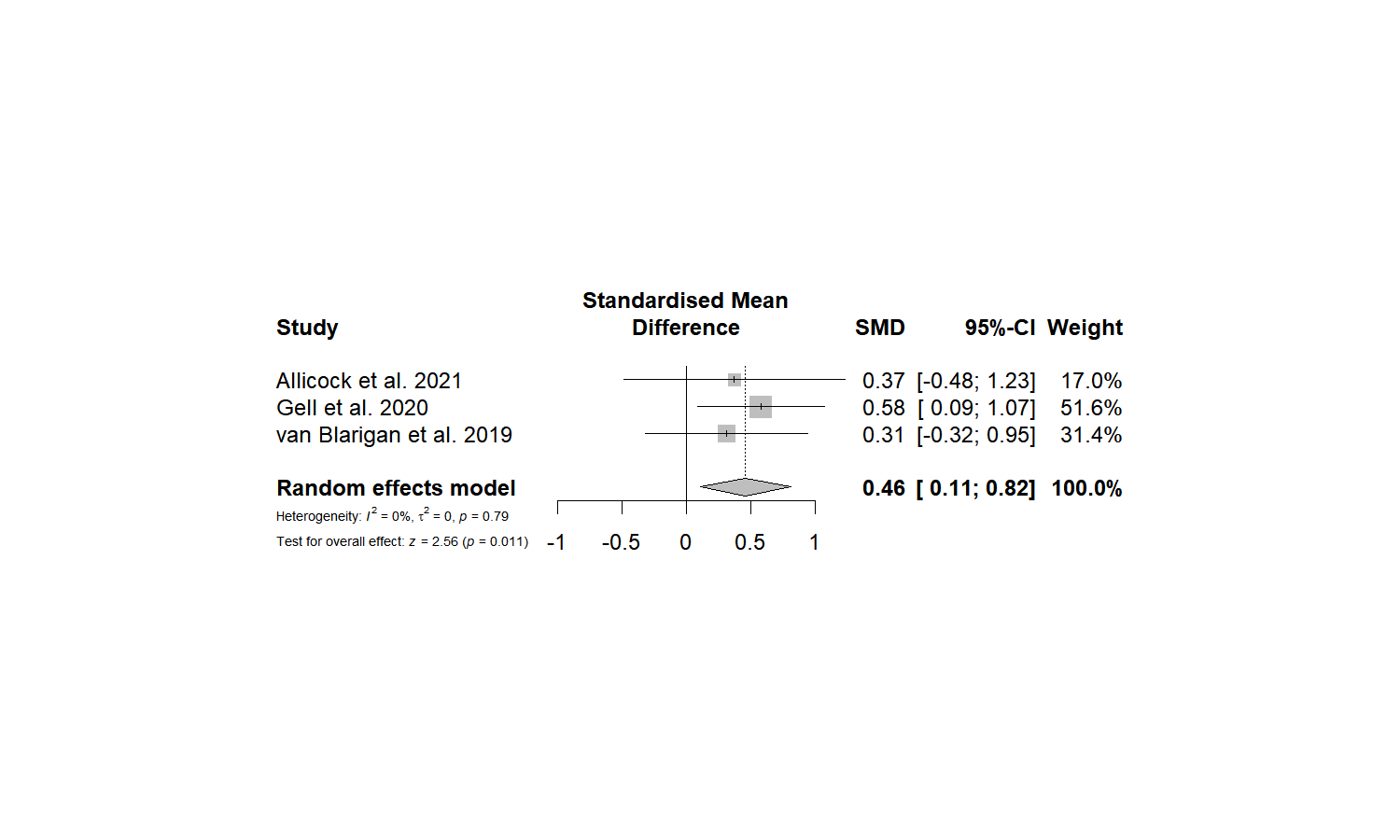

Supplement: Multimedia Appendix 9 [file jmir-v27-e73934-s009.docx]
