# Supplementary material for: Mobile Phone Messaging–Based Interventions to Improve Physical Activity in Patients With Cancer: Systematic Review and Meta-Analysis
Source: J Med Internet Res. 2025 Dec 15;27:e73934. doi: 10.2196/73934 (PMC12704914; doi:10.2196/73934)

Multimedia Appendix 11 Funnel Plots

1. Objective PA at post-intervention


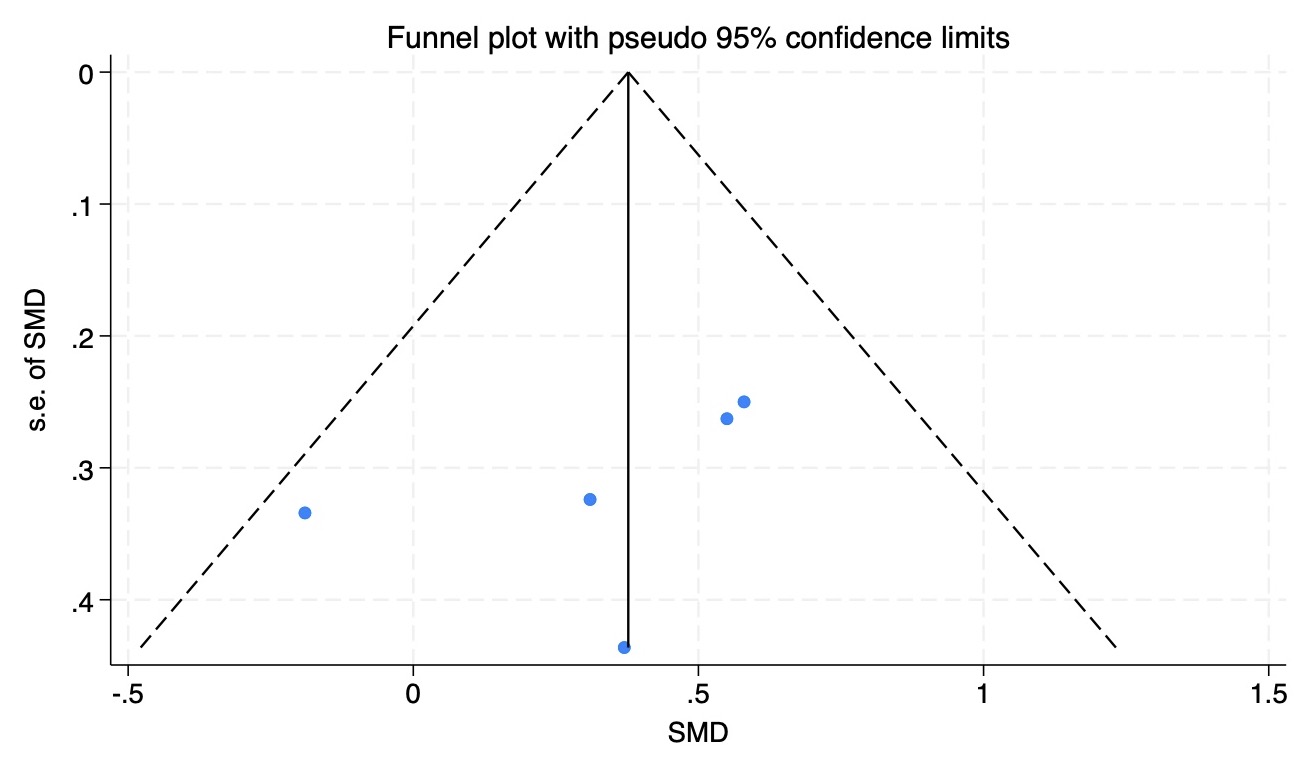


1. Self-reported PA at post-intervention


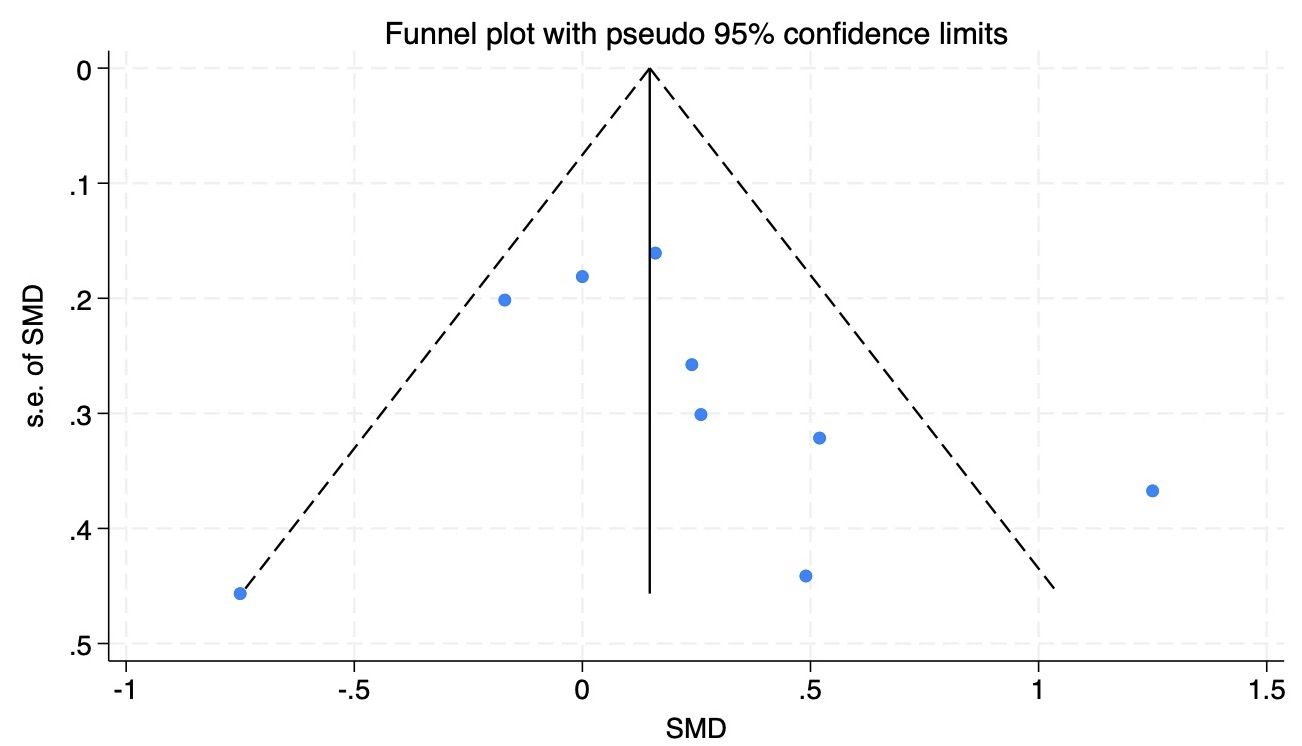


1. Step count at post-intervention


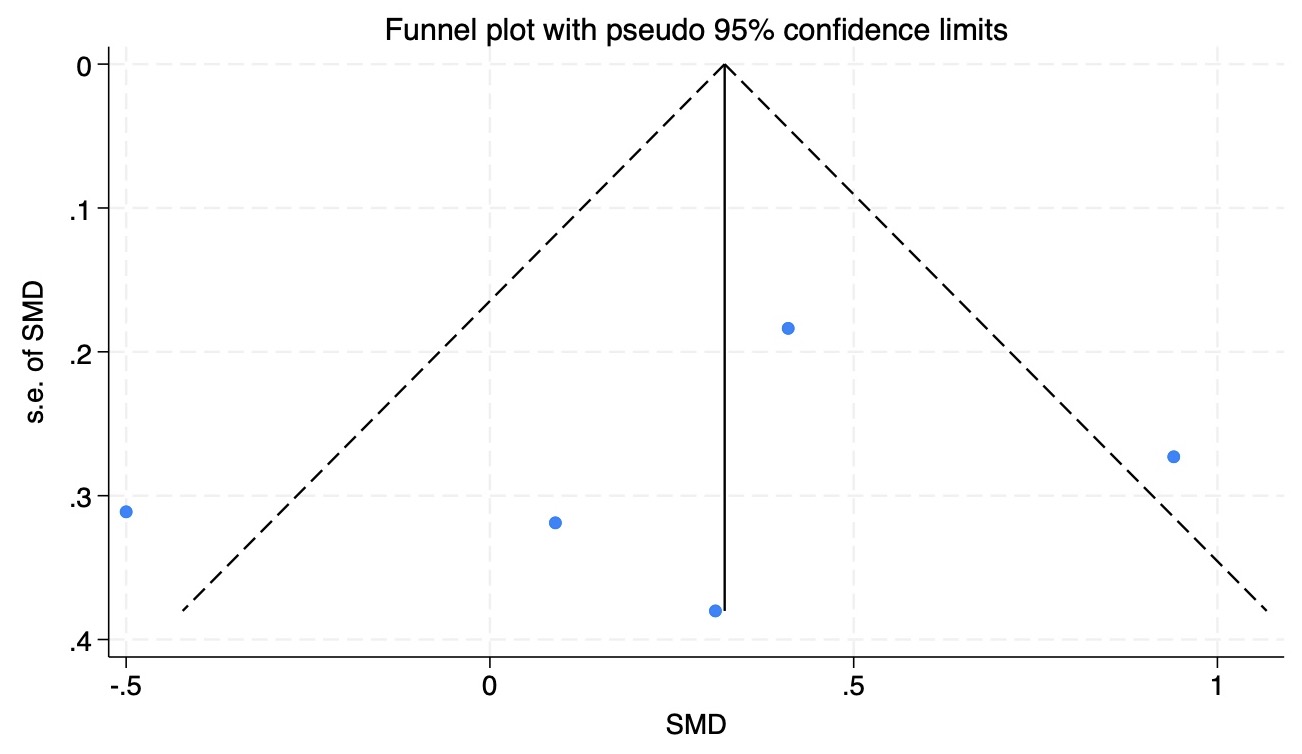


1. Self-reported PA at follow-up


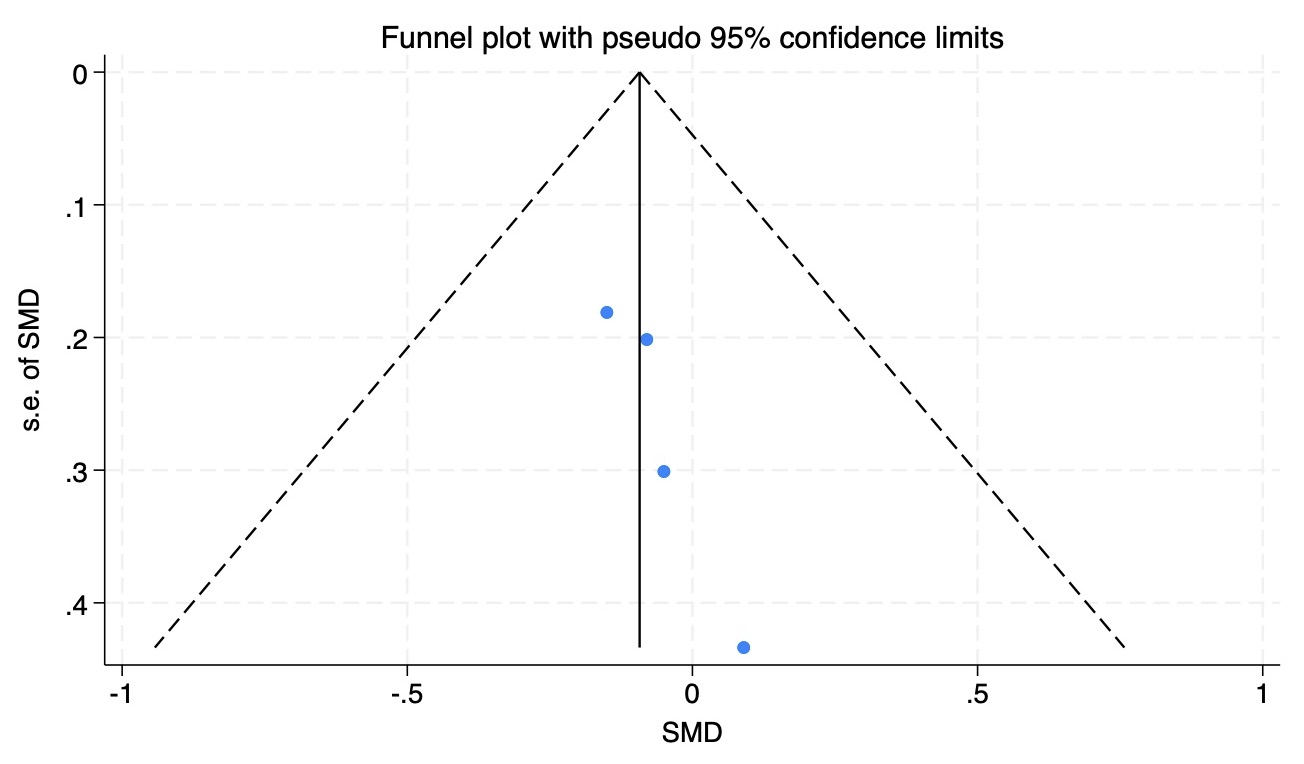

Supplement: Multimedia Appendix 11 [file jmir-v27-e73934-s011.docx]
